# The Influence of MicroRNA-31 on Oxidative Stress and Radiosensitivity in Pancreatic Ductal Adenocarcinoma

**DOI:** 10.3390/cells11152294

**Published:** 2022-07-25

**Authors:** Jason McGrath, Laura E. Kane, Stephen G. Maher

**Affiliations:** Cancer Chemoradiation Research Group, Department of Surgery, Trinity St. James’s Cancer Institute, Trinity Translational Medicine Institute, St. James’s Hospital, D08 W9RT 8 Dublin, Ireland; jmcgrat1@tcd.ie (J.M.); kanela@tcd.ie (L.E.K.)

**Keywords:** microRNA, reactive oxygen species, glutathione peroxidase, oxidative stress, radiotherapy, DNA damage response, pancreatic ductal adenocarcinoma

## Abstract

Radioresistance remains a significant challenge in treating pancreatic ductal adenocarcinoma (PDAC), contributing to the poor survival rates of this cancer. MicroRNAs (miRs) are small non-coding RNA molecules that may play an essential role in regulating radioresistance by altering the levels of oxidative stress. In this study, we investigated the role and potential mechanisms linking miR-31 to PDAC radioresistance. A pCMV-miR vector containing a miR-31 mimic was stably expressed into a miR-31-deficient PDAC cell line, BxPC-3. Additionally, a pmiRZip lentivector suppressing miR-31 was stably expressed in a miR-31 abundant PDAC cell line, Panc-1. Clonogenic assays were conducted to explore the role of miR-31 manipulation on radiosensitivity. Fluorometric ROS assays were performed to quantify ROS levels. The expression of potential miR-31 targets was measured by Western blot analysis. It was found that the manipulation of miR-31 altered the radiosensitivity in PDAC cells by regulating oxidative stress. Using online bioinformatics tools, we identified the 3′UTR of *GPx8* as a predicted target of miR-31. Our study demonstrates, for the first time, that manipulating miR-31 alters GPx8 expression, regulating ROS detoxification and promoting either a radioresistant or radiosensitive phenotype. MiR-31 may represent a promising therapeutic target for altering radiosensitivity in PDAC cells.

## 1. Introduction

Pancreatic cancer is one of the most lethal forms of human malignancy, having a 5-year survival rate of less than 7% [1]. Pancreatic cancer is expected to become the second most common cause of cancer-related death in the United States by 2030 [2], a trend reflected in Europe [3]. Pancreatic ductal adenocarcinoma (PDAC) accounts for over 90% of all pancreatic cancer cases, with surgery being the only curative treatment. The symptoms associated with PDAC, such as abdominal pain or back pain, are notoriously vague and contribute to these cancers’ late diagnosis and subsequent poor survival rates [4]. As a result, only 10–20% of patients are eligible for curative surgery due to late diagnosis [5].

Approximately 35% of patients present with locally advanced PDAC and will receive radiotherapy, an essential component of palliative treatment for patients with metastatic disease [2,5,6]. Unfortunately, tumour resistance to radiotherapy remains a significant clinical challenge in PDAC treatment and is poorly understood [5,6,7]. The features frequently associated with radioresistance include alterations in DNA repair, proliferation, cell-cycle checkpoint control, apoptosis, and altered reactive oxygen species (ROS) biology [8]. As such, elucidating the mechanisms of radioresistance in PDAC is essential for developing new therapeutic approaches to improve treatment efficacy and prolong patient survival.

ROS are unstable oxygen-containing substances that display significant oxidative activity [9]. Radiotherapy generates ROS through the radiolysis of water within the cell [10]. The excessive amounts of ROS induced by radiotherapy account for about two-thirds of the DNA damage caused during treatment, resulting in cell damage and death [11,12]. However, the cell is equipped with an antioxidant defence system that protects against the oxidative damage caused by elevated ROS levels [13]. Nevertheless, alterations within these antioxidant-dense systems have been associated with resistance to radiotherapy by negatively impacting the detoxification of excess ROS [12,13,14].

MicroRNAs (miRs) are small (18–22 nucleotides), non-coding RNAs that regulate gene expression at the post-transcriptional level by predominantly targeting the 3′ untranslated region (UTR) of target mRNAs, resulting in mRNA degradation and inhibition of protein translation [15,16]. Importantly, due to imperfect complementarity, a single miR molecule has the potential to target multiple mRNAs simultaneously, making them attractive therapeutic targets [15,16,17].

One of the key genetic events in PDAC development is the inactivation of the p16 tumour suppressor gene [18]. The p16 gene is encoded on chromosome 9p21.3, a recognised fragile site in the human genome [19]. Interestingly, microRNA-31 (miR-31) is encoded just downstream of p16, and as such, they are frequently co-deleted or co-disrupted together [20]. We have previously demonstrated that miR-31 is a useful therapeutic target that can regulate chemotherapy and radiotherapy sensitivity by altering drug transportation and DNA damage repair genes in other cancer types [21,22]. However, its role in regulating radiosensitivity in PDAC remains to be elucidated.

In this study, we examine the role of miR-31 in radioresistance using PDAC cell lines of differing miR-31 statuses. For the first time, our results show that manipulating miR-31 expression in PDAC cells regulates sensitivity to clinically relevant doses of radiation by targeting an antioxidant enzyme, glutathione peroxidase 8 (GPx8), which plays a vital role in ROS detoxification. We demonstrate miR-31 as a suitable therapeutic target in PDAC through the regulation of sensitivity to radiotherapy via modulation of oxidative stress and DNA damage.

## 2. Materials and Methods

### 2.1. Cell Culture

The PDAC cell lines BxPC-3 and Panc-1 were purchased from ATCC. The cell lines were maintained in an RPMI 1640 medium (Biosciences, San Jose, CA, USA) supplemented with 10% fetal bovine serum (Biosciences) and 1% penicillin/streptomycin (Brennan). The cells were maintained in humidified incubators at 37 °C, with 5% CO_2_. With no apparent contamination, regular mycoplasma testing was carried out using the MycoAlert Mycoplasma Detection Kit (Biosciences).

### 2.2. Radiation Treatment

Irradiation was performed using an X-ray generator (CIX2) (XStrahl) at a dose rate of 1.87 Gray (Gy)/min. Detailed dosimetry and warm-up cycles were performed regularly to ensure the irradiated dose was accurate. The irradiator was also regularly validated and calibrated.

### 2.3. MiR Transfection

According to the manufacturer’s instructions, liposomal transfection was performed using the Lipofectamine 2000 reagent (Invitrogen, Waltham, MA, USA). The MiR-31 overexpression plasmid (MI0000089) and vector control (pCMVMIR) were purchased from Origene (Herford, Germany) and stably transfected into the BxPC-3 cell line under a 400 μg/mL G418 (Gibco) selection for 21 days. The Zip-miR-31 plasmid (MZIP31-PA-1) and Zip vector control (MZIP000-PA-1) were purchased from BioSciences and stably transfected into the Panc-1 cell line under a 2.5 μg/mL puromycin (Sigma Aldrich, St. Louis, MO, USA) selection for ten days.

### 2.4. Reverse Transcription and qPCR

RNA was isolated from cells using the RNeasy Mini kit (QIAGEN Hilden Düsseldorf, Germany ) according to the manufacturers’ instructions. The quantification of RNA was determined by Nanodrop Lite (Thermo Fisher Scientific, Waltham, MA, USA). RNA was reverse transcribed using the QuantiTect Reverse Transcription kit (QIAGEN) according to the manufacturers’ instructions. QuantiTect and the miScript SYBR Green PCR Master Mix (QIAGEN) were used to assess the mRNA and miRNA levels using real-time qPCR (Applied Biosystems, Waltham, MA, USA). Commercially available QIAGEN miScript (miR-31, MS00003290) primer assays were employed, and the RNU6 endogenous control (MS00033740) was used for data normalisation.

### 2.5. Silencing GPx8 in PDAC Cells

The BxPC-3 cells (3 × 10^5^) were transfected with either the siRNA scramble control (4390843) or siRNA *GPx8* (4392420), purchased from Origene, using the Lipofectamine 2000 reagent. The final concentration of siRNA was 10 nM. Transfections were performed using OptiMem (BioSciences), and the cells were treated 48 h post-transfection.

### 2.6. Clonogenic Assay

Clonal survival was determined by seeding the optimal density of cells (1 × 10^3^–5 × 10^3^) into 6-well plates and adhering overnight at 37 °C in 5% CO_2_/95% humidified air. The spent medium was discarded, and a fresh medium was applied before the radiation treatment. The plates were then incubated for 8–10 days after seeding. The colonies were fixed and stained with crystal violet solution (0.1% *w*/*v* crystal violet, 60% *v*/*v* methanol, 40% *v*/*v* deionized water), and the wells were washed with water until the colonies were distinctive. The colonies were counted using a GelCount instrument (Oxford Optronix, Ltd., Oxford, UK) and optimised via the compact Hough and radial map (CHARM) image processing algorithms for each cell line. The plating efficiencies (PE) were calculated using the formula: PE = number of colonies counted/number of cells seeded. The surviving fraction (SF) was calculated using the formula: SF = number of colonies counted/PE × number of cells seeded.

### 2.7. Cumulative Proliferation Assay

A proliferation assay was employed, wherein 3 × 10^5^ cells were seeded into 10 cm^2^ tissue culture dishes and allowed to adhere overnight. The cells were treated with 4 Gy radiation. The cells were reseeded at 3 × 10^5^ every three days for nine days, and a cumulative cell count was taken.

### 2.8. Measurement of Intracellular ROS

H_2_O_2_ was measured using the fluorometric-near infrared ROS assay kit (Abcam, Cambridge, UK). The cells were seeded at a concentration of 1 × 10^4^ cells/well into an opaque 96-well plate and were allowed to adhere overnight at 37 °C in 5% CO_2_/95% humidified air. The cells were treated with radiation and returned to the incubator for the appropriate time interval. Briefly, an H_2_O_2_ reaction mixture was prepared according to the manufacturer’s instructions. A 50 µL volume of the H_2_O_2_ reaction mixture was added to each well and incubated at room temperature for 0–30 min, protected from light. Fluorescence was measured at Ex/Em = 640/680 nm using a GloMax microplate reader (Promega, Southampton, UK).

### 2.9. Measurement of Intracellular GSH/GSSG Levels

Levels of reduced glutathione (GSH) were measured using the luminescence-based GSH/GSSG-Glo^TM^ assay (Promega). The cells were seeded at a concentration of 5 × 10^3^ cells/well into a white-bottomed 96-well plate and were allowed to adhere overnight at 37 °C in 5% CO_2_/95% humidified air. The cells were treated with appropriate treatment for 24 h. After treatment, the spent medium was discarded to waste, and 50 µL of total GSH was applied to each well and mixed using an orbital shaker (Medical Supply Co., Dublin, Ireland) for 5 min. A volume of 50 µL luciferase generation reagent was added to all wells, and the plates were incubated for 30 min. A volume of 100 µL of luciferase detection reagent was added to all wells and incubated for 15 min at room temperature. Finally, the luminescence was read with the GloMax microplate reader (Promega), with an integration time of 1000 ms.

### 2.10. Measurement of Caspase 3/7 Levels

Caspase 3/7 activity was measured using the ApoTox-Glo^TM^ assay (Promega). The cells were seeded at a concentration of 1 × 10^4^ cells/well into a white-bottomed 96-well plate and were allowed to adhere overnight at 37 °C in 5% CO_2_/95% humidified air. The cells were treated with radiation and left for appropriate time intervals. A volume of 100 µL of caspase 3/7 substrate, dissolved in caspase 3/7 buffer, was added to each well. The plates were placed on the orbital shaker for 30 s, left to incubate for 20 min at room temperature, and were finally measured for the luminescence signal using a GloMax microplate reader (Promega) with a 1000 ms integration time.

### 2.11. Protein Extraction and Western Blot Analysis

Total protein was extracted using the cold radioimmunoprecipitation assay (RIPA) buffer (50 mM Tris-HCl (pH 8), 1% Triton X, 0.5% sodium deoxycholate, 0.1% SDS, 150 mM NaCl) with the addition of protease and phosphatase inhibitor tablets (Roche, Rotkreuz, Switzerland). Lysates were centrifuged at 500× *g* for 10 min, and the supernatant was collected for analysis. The protein concentration was quantified using the BCA protein assay kit (Thermo Fisher Scientific), with 30 µg of protein per sample loaded onto gels. The proteins were separated on 6-12% SDS-PAGE gels, transferred onto polyvinylidene fluoride (PVDF) membrane (Thermo Scientific), and probed for gamma-H2A.X (S139) (Cell Signal) used at a 1:2000 dilution, GPx8 (CAB20390) (Reagent Genie Dublin 2 Ireland) used at a 1:2000 dilution, and beta-actin (sc-69879) (Biotechnology, Santa Cruz, CA, USA) used at a 1:10,000 dilution, followed by incubation with anti-mouse horseradish peroxidase (HRP)-conjugated secondary antibody (7074S) (Cell Signal) used at a 1:2000 dilution, or anti-rabbit HRP-conjugated secondary antibody (7076S) (Cell Signal, Boston, MA, USA) used at a 1:2000 dilution. Bands were detected using the SuperSignal^TM^ West Pico PLUS chemiluminescence substrate (Thermo Fisher, Gloucester, UK) and visualised using the FusionFx imager (Vilber). The densitometric analysis was performed with the Image lab software 1.0 (Bio-Rad, Hercules, CA, USA). The volume intensity of a band was normalised to the volume density of the loading control, beta-actin, by dividing the band of interest by the beta-actin band. Where appropriate, the volume densities of bands were then normalised to the densities of a control sample.

### 2.12. Statistical Analysis

Experiments were repeated at least three times, and the results displayed as mean ± SEM. The statistical significance of the results was determined by a two-tailed paired *t*-test, a one-sample *t*-test, and a one/two-way ANOVA; * *p* < 0.05 was considered a statistically significant difference.

## 3. Results

### 3.1. Establishing a Stable miR-31 Model in PDAC Cell Lines

**Figure 1:**
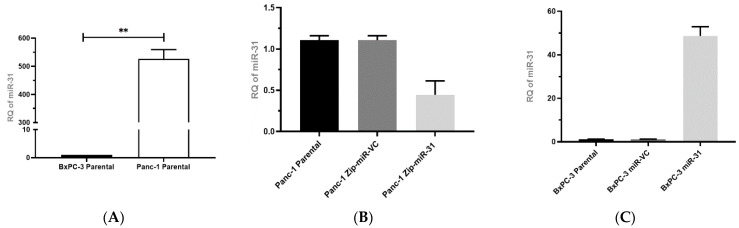
Establishing and confirming miR-31 stable transfection in PDAC cell lines. (**A**) qPCR evaluating the relative level of miR-31 expression between BxPC-3 and Panc-1 cell lines. Endogenous miR-31 were significantly higher in Panc-1 cells compared to BxPC-3 cells (** *p* = 0.0038). Data are expressed as the mean ± SEM and analyzed by a one-sample *t*-test (*n* = 3). (**B**) qPCR evaluating the relative levels of miR-31 expression between Panc-1 parental, Zip-miR-VC, and Zip-miR-31 cell lines (*n* = 3). (**C**) RT-qPCR evaluating the relative levels of miR-31 expression between BxPC-3 Parental, miR-VC, and miR-31 cell lines (*n* = 3).

The relative expression of miR-31 was substantially lower in the BxPC-3 cell line compared to the Panc-1 cell line (Figure 1A). Following the transfection of BxPC-3 cells with either the miR-VC or miR-31 expressing plasmids and the transfection of Panc-1 cells with the suppression plasmids Zip-miR-VC or Zip-miR-31, confirmation of miR-31 overexpression or suppression in the stable expressing models were measured by qPCR (Figure 1B,C). The Panc-1 Zip-miR-31 cell line displayed a successful transfection by presenting a reduced RQ of miR-31 compared to its vector control equivalent. Similarly, the BxPC-3 miR-31 cell line showed a successful transfection by presenting a greater RQ of miR-31 than its vector control equivalent. Additionally, to confirm the expression of the miR-VC or Zip-miR-VC within cells, an analysis of the GFP reporter was undertaken via Western blot (results not shown).

### 3.2. Manipulating miR-31 Regulates Radiosensitivity in PDAC Cell Lines

**Figure 2:**
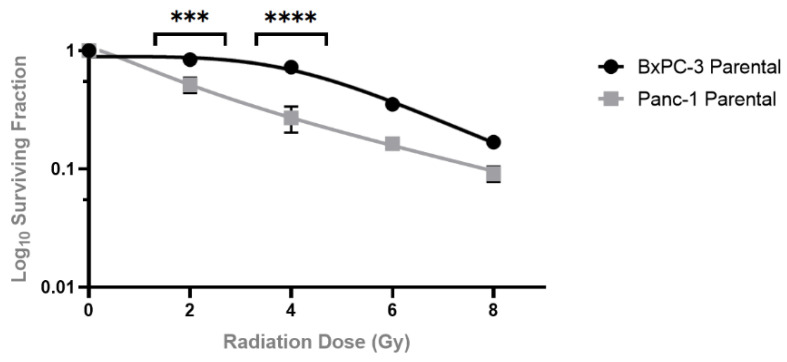
Clonogenic survival of PDAC cell lines when treated with radiotherapy. BxPC-3 and Panc-1 parental cell lines were irradiated with 2 Gy, 4 Gy, 6 Gy, and 8 Gy. Control cells were mock-irradiated (0 Gy). Data are represented as the mean ± SEM (*n* = 3). Two-way ANOVA with Tukey’s post-hoc test was performed for statistical analysis, comparing BxPC-3 cells to Panc-1 cells at 2 Gy (*** *p* = 0.0010), 4 Gy (**** *p* < 0.0001), 6 Gy *p* = 0.1213) and 8 Gy (*p* = 0.9495).

**Figure 3:**
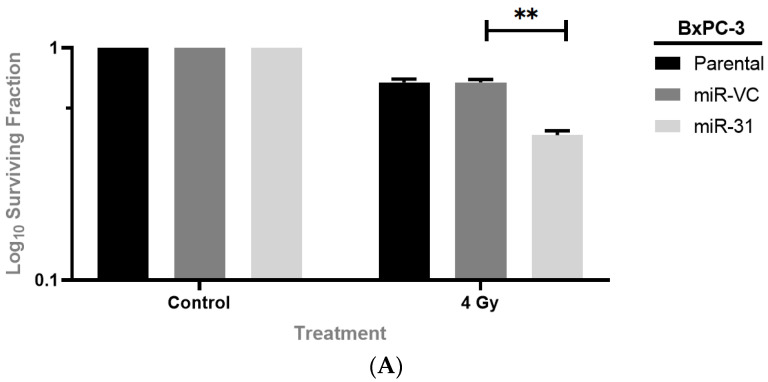

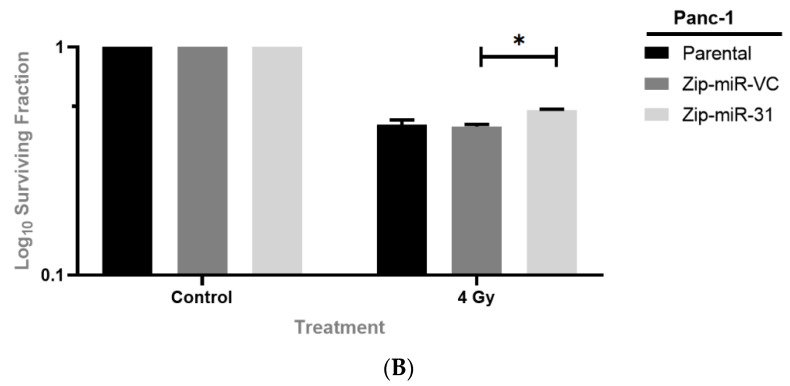
Manipulating miR-31 regulates radiosensitivity in PDAC cell lines. (**A**) Overexpressing miR-31 in BxPC-3 cells significantly reduced surviving fraction (** *p* = 0.00891) post-irradiation. (**B**) Suppressing miR-31 in Panc-1 cells showed a significant increase in survival fraction (* *p* = 0.0211). All cells were irradiated with 4 Gy, while controls were mock-irradiated (0 Gy) 24 h post-seeding. Data are expressed as the mean ± SEM and analyzed by a two-tailed paired *t*-test (*n* = 3).

Clonogenic assays were used to evaluate whether manipulating miR-31 regulates sensitivity to the clinically relevant doses of radiation (2 Gy to 8 Gy) in PDAC cells. The BxPC-3 parental cells displayed a more radioresistant phenotype when compared to the Panc-1 parental cells, with significant differences seen at the 2 Gy and 4 Gy doses of radiation (Figure 2). For the subsequent experiments, all cell lines were treated with 4 Gy radiation, as it provided a reasonable margin to determine the effect of miR-31 on cell survival. The clonogenic assays revealed that overexpressing miR-31 in BxPC-3 cells significantly enhanced sensitivity to radiation treatment (Figure 3A), with a significant reduction in the surviving fraction observed compared to its vector control equivalent. Conversely, suppressing miR-31 in Panc-1 cells promoted resistance to radiation treatment, with a modest yet significant increase in the surviving fraction observed (Figure 3B). Irradiation with 4 Gy significantly increased the survival fraction (* *p* = 0.0211) when suppressing miR-31 in Panc-1 cells compared to its vector control equivalent.

**Figure 4:**
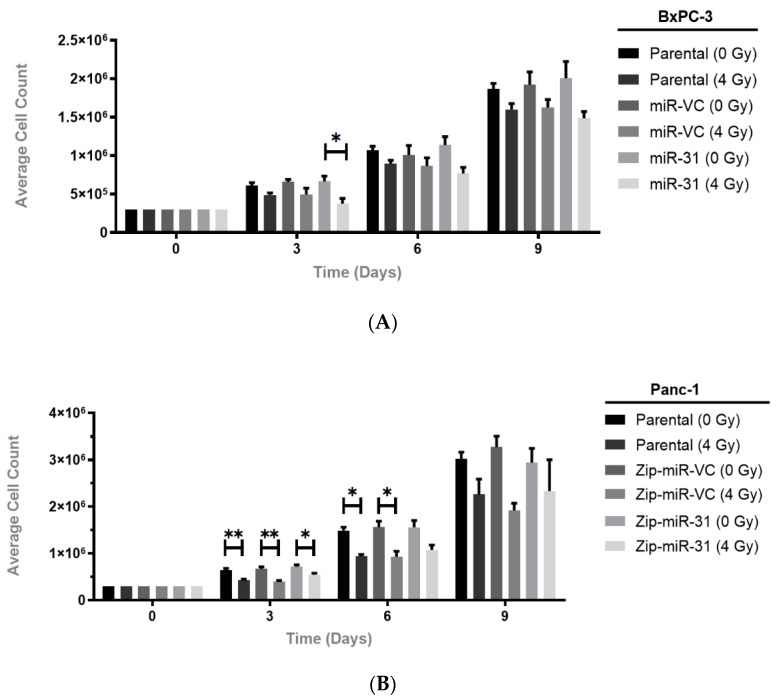
Manipulating miR-31 alters cell proliferation post-radiation treatment. (**A**) Assaying cumulative proliferation with radiation treatment revealed no significant decrease in cell count at any time points measured in BxPC-3-miR-VC irradiated cells (4 Gy) compared to mock-irradiated cells (0 Gy). MiR-31-expressing irradiated cells displayed a significant decrease in cell count at day 3 (* *p* = 0.0353) compared to their mock-irradiated counterpart. (**B**) A significant reduction in cell count was displayed at day 3 (** *p* = 0.00122) and day 6 (* *p* = 0.0120) in Panc-1 Zip-miR-VC irradiated cells compared to mock-irradiated cells, while Zip-miR-31 irradiated cells displayed a reduction in cell count at day 3 only (* *p* = 0.0354) compared to mock-irradiated cells. Data are expressed as the mean ± SEM (*n* = 3). Two-way ANOVA with Tukey’s post-hoc test was adopted for statistical analysis.

Following on from the observation that suppressing miR-31 increased clonogenic survival in the Panc-1 cells and overexpressing miR-31 decreased survival in the BxPC-3 cells, following irradiation, a cumulative cell count was undertaken to determine whether miR-31 alone, without the influence of radiotherapy, would affect proliferation. It is well-established that proliferation rates can influence radiosensitivity, where cells with a higher proliferation rate are more radiosensitive than more slowly proliferating cells. It was observed that miR-31 manipulation without the influence of radiotherapy produced no significant change in the proliferation rate (Figure 4). Subsequently, while no significant differences in proliferation at any time points were observed in the BxPC-3-miR-VC cells following irradiation, the miR-31-overexpressing cells appeared more sensitive to radiation treatment on day 3 (* *p* = 0.0353) by displaying a reduction in cell count (Figure 4A). Assaying cumulative proliferation with radiation treatment revealed a significant reduction in proliferation at day 3 (** *p* = 0.00122) and day 6 (* *p* = 0.0120) in the Panc-1 Zip-miR-VC cells, whereas the Zip-miR-31 cells appeared less sensitive to the radiation treatment at day 3 (* *p* = 0.0354) and day 6 (*p* = 0.0662) (Figure 4B). Overall, miR-31 alone does not alter cell proliferation, as assessed by a cumulative proliferation assay. However, after radiation treatment, miR-31 encouraged a reduced cell count, possibly explaining the differences in clonogenicity.

### 3.3. Manipulating miR-31 Alters DNA Damage Induction and Repair in PDAC Cell Lines

**Figure 5:**
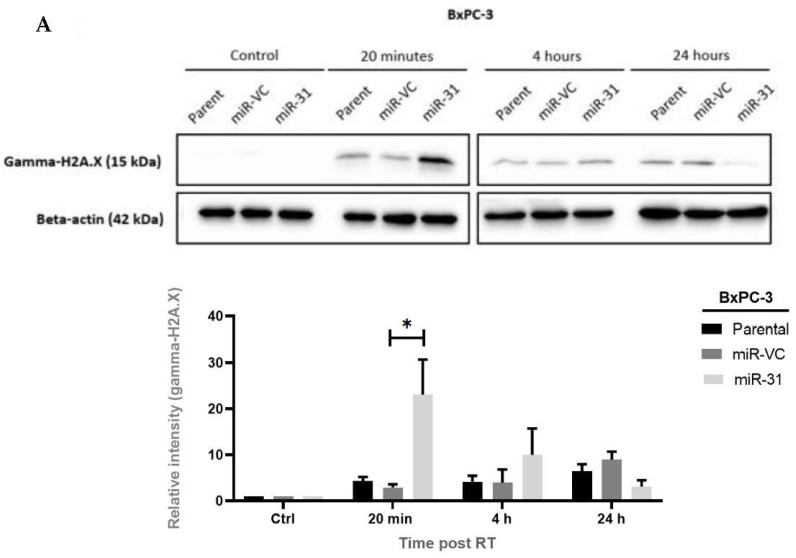

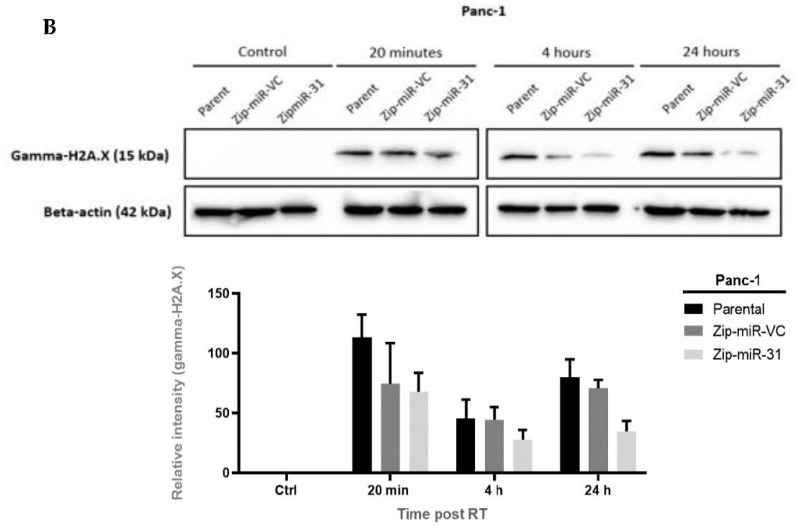
The expression of miR-31 positively correlates with DNA damage incurred when treated with radiation. (**A**) Representative Western blot time course and densitometric analysis for gamma-H2A.X as a marker of DNA damage following radiation treatment (RT) in BxPC-3 cells. Significant increases in gamma-H2A.X levels were observed in the BxPC-3 miR-31 cells 20 min (* *p* = 0.0120) post-RT. Interestingly, levels of gamma-H2A.X were reduced, with no significant differences observed at 4 h (*p* = 0.932) and 24 h (*p* = 0.939) post-RT. (**B**) Representative Western blot time course and densitometric analysis for gamma-H2A.X as a marker of DNA damage with RT (4 Gy) in Panc-1 cells. It is evident that levels of gamma-H2A.X decreased in Panc-1 Zip-miR-31 cells, however, no significant differences were observed at 20 min (*p* > 0.999), 4 h (*p* = 0.990), and 24 h post-RT (*p* = 0.664). Data are represented as the mean ± SEM (*n* = 3). Two-way ANOVA with Tukey’s post-hoc test was adopted for statistical analysis.

Radiation-induced cell death is frequently due to DNA damage, especially to double-strand DNA breaks (DSBs), and alterations in the DNA repair systems have been strongly associated with radioresistance. Having observed the differences in clonogenic survival, we examined the influence of miR-31 on DNA damage induction and repair by investigating the levels of gamma-H2A.X, which occurs at the sites of DSBs. We found that overexpressing miR-31 in BxPC-3 cells significantly increased the levels of gamma-H2A.X 20 min post-radiation treatment (* *p* = 0.0120), whereas the levels of gamma-H2A.X are reduced at 4 h *(p* = 0.932) and 24 h (*p* = 0.939) post-radiation (Figure 5A). Gamma-H2A.X levels were shown to be decreased in Panc-1 Zip-miR-31 cells; however, no significant differences were observed at 20 min (*p* > 0.999), 4 h (*p* = 0.990), or 24 h post-radiation treatment (*p* = 0.664), despite a trend being observed (Figure 5B). Subsequently, to determine if the levels of DNA damage corresponded to cell death, apoptosis was assessed post-radiation treatment.

### 3.4. Manipulating miR-31 Alters Radiation-Induced Apoptosis in PDAC Cell Lines

**Figure 6:**
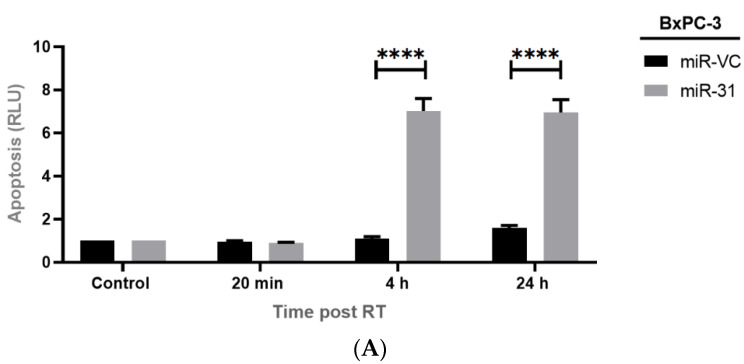

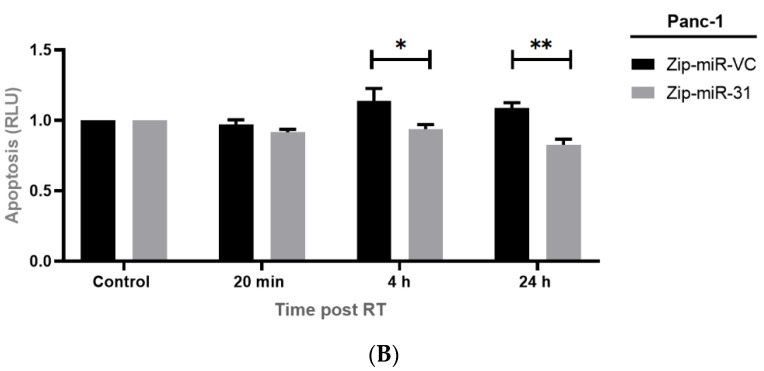
Manipulating miR-31 alters apoptosis in PDAC cells. Caspase 3/7 activity was measured as a marker of apoptosis at 20 min, 4 h, and 24 h post-radiation treatment (RT). (**A**) There were no significant differences in apoptosis in 20 min post-RT (*p* > 0.999) between BxPC-3 miR-VC and BxPC-3 miR-31 cells. However, overexpressing miR-31 in BxPC-3 cells displayed a significant increase in apoptosis 4 h (**** *p* < 0.0001) and 24 h (**** *p* < 0.0001) post-RT. (**B**) There are no significant differences in apoptosis 20 min post-RT between Panc-1 Zip-miR-VC and Panc-1 Zip-miR-31 cells (*p* = 0.9368). Although suppressing miR-31 in Panc-1 cells displayed a significant decrease in apoptosis 4 h (* *p* = 0.0199) and 24 h (** *p* = 0.00630) post-RT. Data are represented as the mean ± SEM (*n* = 3). Two-way ANOVA with Tukey’s post-hoc test was adopted for statistical analysis.

To study a possible cause of cell sensitivity to radiation treatment, we measured caspase 3/7 activity as a marker of apoptosis. Overexpressing miR-31 in BxPC-3 cells displayed no significant changes in caspase 3/7 at 20 min (*p* > 0.999) post-radiation treatment. However, a significant increase in caspase 3/7 activity was observed at 4 h (**** *p* < 0.0001) and 24 h (**** *p* < 0.0001) post-radiation treatment (Figure 6A). Suppressing miR-31 in Panc-1 cells displayed no significant differences at 20 min post-radiation treatment (*p* = 0.968). However, a significant reduction in caspase 3/7 activity was observed at 4 h (* *p* = 0.0498) and 24 h (** *p* = 0.001) post-radiation treatment (Figure 6B).

### 3.5. Manipulating miR-31 Alters Reactive Oxygen Species (ROS) Levels in PDAC Cell Lines

**Figure 7:**
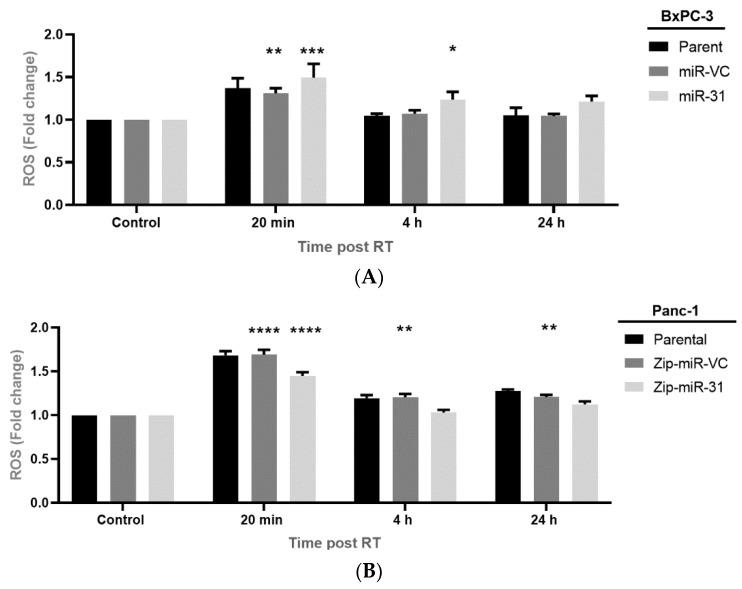
Manipulating miR-31 alters ROS levels in PDAC cell lines. (**A**) ROS levels were assessed 20 min, 4 h, and 24 h post-radiation treatment (RT) and compared to the untreated control (0 Gy). Overexpressing miR-31 in BxPC-3 cells resulted in a significant increase in ROS levels 20 min (*** *p* < 0.001) and 4 h (* *p* = 0.0295) post-RT. Meanwhile, miR-VC cells displayed a significant increase in ROS levels at 20 min only (** *p* = 0.0018) post-RT, which had returned to baseline levels 4 h and 24 h post-RT. (**B**) Similarly, ROS levels were assessed 20 min, 4 h, and 24 h post-radiation treatment (RT) and compared to the untreated control (0 Gy). In miR-31-suppressed Panc-1 cells, ROS levels significantly increased 20 min (**** *p* < 0.0001) post-RT but returned to baseline 4 h and 24 h post-RT. Conversely, Panc-1 Zip-miR-VC cells displayed a significant elevation in ROS levels 20 min (**** *p* < 0.0001), 4 h (** *p* = 0.0086) and 24 h (** *p* = 0.0067) post-RT. Data are expressed as the mean ± SEM (*n* = 3). Two-way ANOVA with Tukey’s post-hoc test was adopted for statistical analysis.

To determine whether the ROS levels contributed to DNA damage and potentially radioresistance within our models, we analyzed ROS generation 20 min, 4 h, and 24 h post-radiation treatment and compared this to its untreated cells. We found that overexpressing miR-31 in BxPC-3 cells resulted in a significant increase in ROS generation when treated with 4 Gy compared to its untreated control at 20 min (**** *p* < 0.0001) and 4 h (* *p* = 0.0295), while no significant increase was observed 24 h post-radiation treatment (*p* = 0.0690). The vector control equivalent displayed a significant increase in ROS generation at 20 min (** *p* = 0.00180) post-radiation treatment only (Figure 7A). Furthermore, suppressing miR-31 in Panc-1 cells resulted in a significant increase in ROS generation when treated with 4 Gy compared to the untreated control at 20 min (**** *p* < 0.0001) post-radiation treatment, but no significant increase was displayed at 4 h ^(^*p* > 0.999) post-radiation treatment. Moreover, a significant increase was observed in the vector control equivalent at 20 min (**** *p* < 0.0001), 4 h (** *p* = 0.00860) and 24 h (** *p* = 0.00670) post-radiation treatment (Figure 7B). Overall, these data indicate a role for miR-31-monitored ROS generation post-radiation treatment, subsequently impacting downstream DNA damage. A possible explanation for this is that miR-31 is altering the levels of antioxidants, which are essential for scavenging ROS, resulting in their detoxification and elimination.

### 3.6. Manipulating miR-31 Does Not Alter Glutathione (GSH) Levels in PDAC Cell Lines

**Figure 8:**
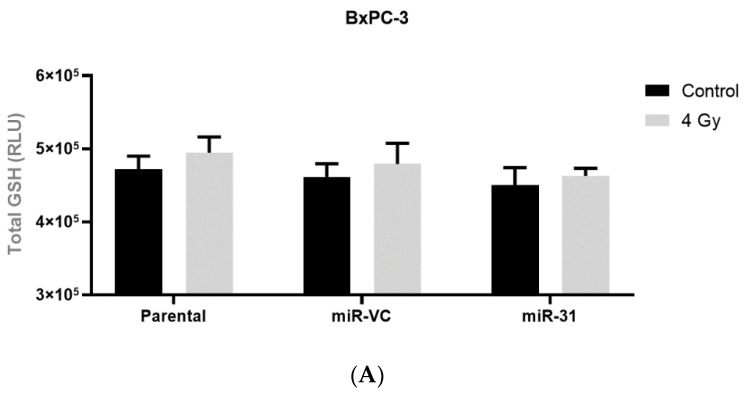

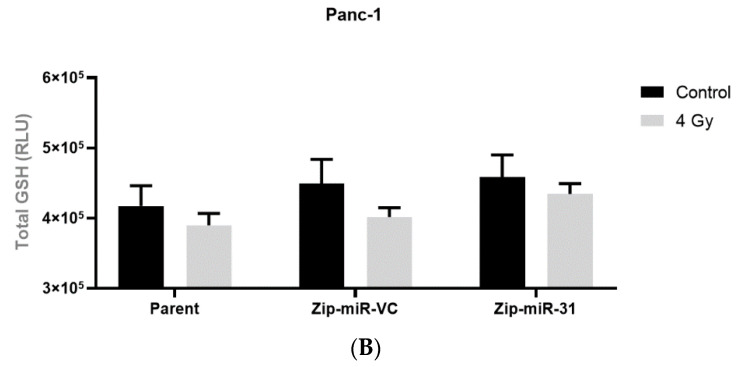
Manipulating miR-31 does not alter total glutathione (GSH) levels in PDAC cell lines. Total GSH in (**A**) BxPC-3 parental, miR-VC, and miR-31 overexpressing cells, and (**B**) Panc-1 parent, Zip-miR-VC and Zip-miR-31 suppressed cells, post-radiation with 0 Gy and 4 Gy. No significant differences were observed. Data are expressed as the mean ± SEM and were analyzed by a two-tailed paired *t*-test (*n* = 3).

The glutathione (GSH) levels were assessed 24 h post-radiation treatment. We observed no significant changes in the GSH between the treated and untreated cells within both the BxPC-3 (Figure 8A) and Panc-1 (Figure 8B) models.

### 3.7. Overexpressing miR-31 Alters Glutathione Peroxidase 8 (GPx8) in PDAC Cell Lines

**Figure 9:**
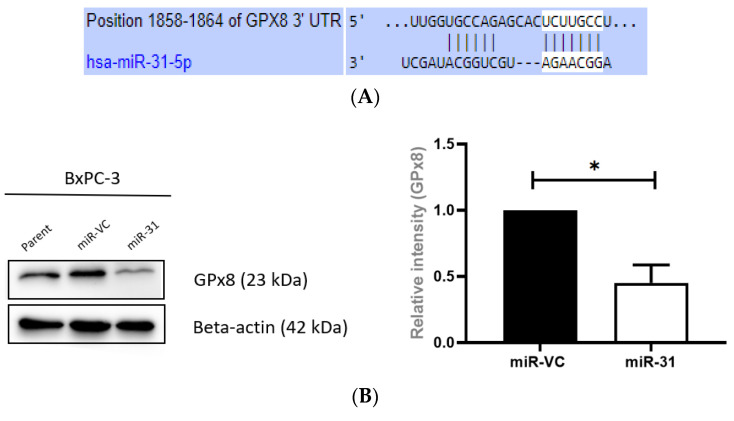

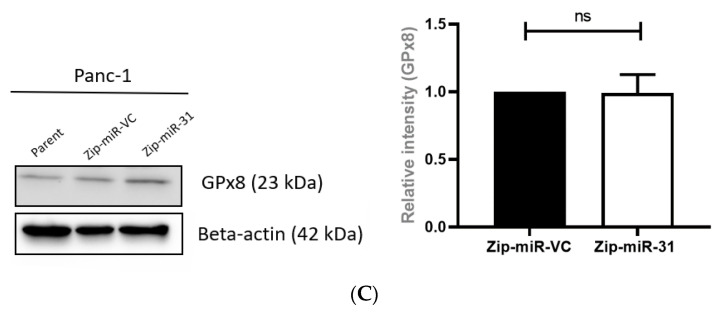
Manipulating miR-31 in PDAC cells alters the expression of GPx8. (**A**) The putative binding site for miR-31 in the 3′UTR of GPx8. (**B**) Representative Western blot illustrating GPx8 levels in BxPC-3 cells. Overexpressing miR-31 in BxPC-3 cells significantly reduced GPx8 levels compared to its vector control equivalent (* *p* = 0.0279). (**C**) Representative blot illustrating GPx8 levels in Panc-1 models. Suppressing miR-31 in Panc-1 cells does not significantly alter GPx8 levels (^ns^ *p* = 0.947) compared to its vector control equivalent. Data are expressed as the mean ± SEM and were analyzed by a one-sample *t*-test (*n* = 3).

The potential regulation of sensitivity to radiation treatment by miR-31 is attributed to its ability to alter the expression of its target genes. The miR target prediction algorithms TargetScan (http://www.targetscan.org/vert_72/, accessed on 18 May 2022), miRTargetLink (https://ccb-web.cs.uni-saarland.de/mirtargetlink/), and miRWalk (http://mirwalk.umm.uni-heidelberg.de/, accessed on 18 May 2022) predicted that the 3′UTR of *GPx8* mRNA contained putative miR-31 binding sites (Figure 9A). To determine whether miR-31 regulates the radiosensitivity of PDAC cells by altering GPx8, the levels of GPx8 in the PDAC models were quantified by Western blot. It was found that overexpressing miR-31 resulted in a significantly reduced GPx8 expression in BxPC-3 cells (* *p* = 0.0279) (Figure 9B). Conversely, suppressing miR-31 in Panc-1 cells displayed a modest (but not statistically significant) increase in GPx8 expression (Figure 9C).

### 3.8. Silencing GPx8 Enhances Radiosensitivity in BxPC-3 Cells

**Figure 10:**
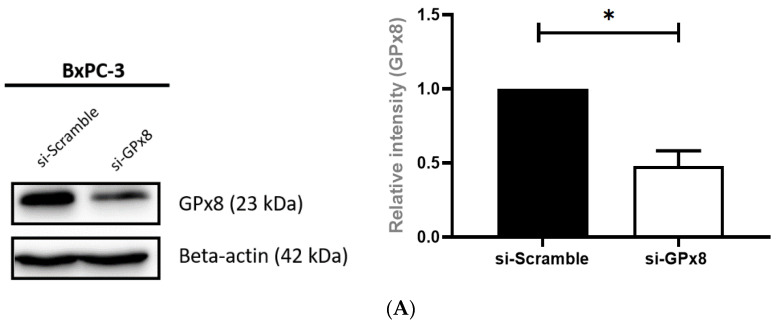

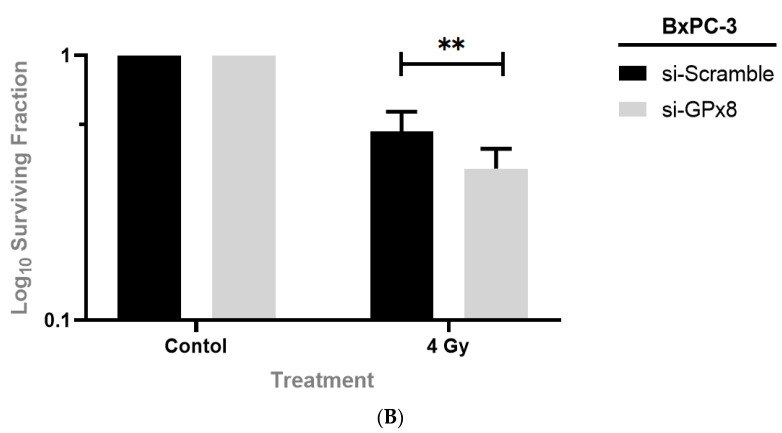
Silencing GPx8 in BxPC-3 cells enhances sensitivity to radiation treatment. (**A**) Representative Western blot confirming GPx8 silencing in BxPC-3 cells. BxPC-3 cells were transiently transfected with either si-Scramble or si-GPx8 for 48 h. Densitometric analysis revealed a significant reduction in GPx8 expression (* *p* = 0.0434) in si-GPx8 cells compared to si-Scramble cells. Data are presented as the mean ± SEM and analyzed by a one-sample *t*-test (*n* = 4). (**B**) Clonogenic analysis revealed that silencing GPx8 in BxPC-3 cells significantly reduced the surviving fraction (** *p* = 0.00353) when compared to its scrambled control. All cells were irradiated with 4 Gy whilst controls were mock-irradiated (0 Gy) 48 h post-transfection. Data are expressed as the mean ± SEM and were analyzed by a two-tailed paired *t*-test (*n* = 7).

With a correlation between the overexpressing miR-31 and GPx8 downregulation, possibly modulating radiosensitivity, further experiments were performed to elucidate if the GPx8 modification alone was sufficient for radiosensitizing PDAC cells. This was investigated by silencing GPx8 in BxPC-3 parental cells, independent of miR-31 modification. GPx8 silencing was confirmed by Western blot (Figure 10A). The clonogenic analysis revealed that silencing GPx8 significantly reduced the surviving fraction (** *p* = 0.00353) post-radiation treatment compared to its scrambled control (Figure 10B), indicating an influence of GPx8 on radiosensitivity in PDAC cells.

### 3.9. Silencing GPx8 Alters Reactive Oxygen Species (ROS) in BxPC-3 Cells

**Figure 11:**
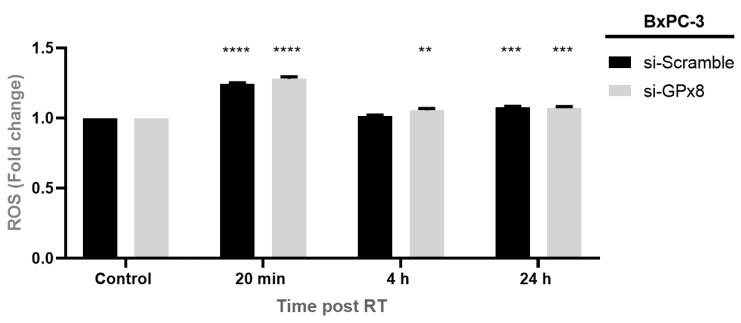
Silencing GPx8 alters ROS levels post-radiation treatment. ROS levels were assessed 20 min, 4 h, and 24 h post-radiation treatment (RT) and compared to its untreated control (0 Gy). Radiation treatment resulted in a significant increase in ROS levels at 20 min (**** *p* < 0.0001), 4 h (** *p* = 0.0073), and 24 h (*** *p* = 0.0004) post-RT. Levels of ROS returned to baseline by 4 h post-RT in the scrambled control, but remained elevated in cells where GPx8 had been silenced. Data are expressed as the mean ± SEM (*n* = 3). Two-way ANOVA with Tukey’s post-hoc test was adopted for statistical analysis.

To determine if GPx8 altered ROS levels and, thus, the radiosensitivity in PDAC cells, we silenced GPx8 in the BxPC-3 parental cells and assessed the ROS levels at 20 min, 4 h, and 24 h post-radiation treatment (Figure 11). We show that silencing GPx8 resulted in a significant increase in ROS levels at 20 min (**** *p* < 0.0001) when compared to its untreated control (0 Gy). Similarly, ROS levels were significantly increased in the scrambled control cells at 20 min post-radiation treatment (**** *p* < 0.0001). Interestingly, ROS levels were still significantly increased when silencing GPx8 at 4 h (** *p* = 0.0073) post-radiation treatment, but no significant changes were observed in the scrambled control cells at 4 h (*p* = 0.934) post-radiation treatment. A significant increase in ROS levels was observed at 24 h post-radiation in the si-GPx8 cells (*** *p* = 0.0004) and the si-Scramble equivalent (*** *p* = 0.0002).

### 3.10. GPx8 Protects BxPC-3 Cells against DNA-Damage Post-Radiation Treatment

**Figure 12:**
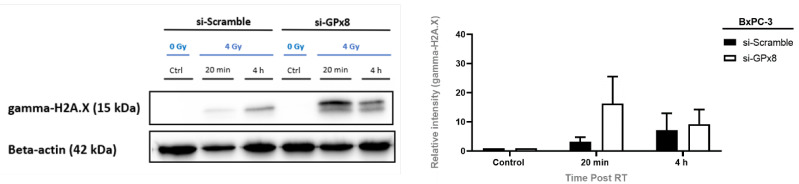
Silencing GPx8 increases gamma-H2A.X levels post-radiation treatment. Representative Western blot time course and densitometric analysis for gamma-H2A.X as a marker of DNA damage with radiation treatment (RT) in si-GPx8 and si-Scramble BxPC-3 cells (*n* = 3). A trend indicated that gamma-H2A.X levels were increased at 20 min post-RT in BxPC-3 cells with GPx8 silenced compared to its scramble control. However, no statistical significance was found. Two-way ANOVA with Tukey’s post-hoc test was adopted for statistical analysis, comparing si-GPx8 BxPC-3 cells to si-Scramble BxPC-3 cells at 20 min post-RT and 4 h post-RT.

As GPx8 expression was associated with radioresistance, potentially by promoting ROS detoxification compared to the cells with lower GPx8 levels, gamma-H2A.X was assessed at 20 min and 4 h post-radiation treatment to determine if silencing GPx8 affected DNA damage (Figure 12). A trend indicated that silencing GPx8 in BxPC-3 cells increased the gamma-H2A.X levels compared to its scrambled control 20 min post-radiation treatment. However, no statistical significance was observed. Nevertheless, GPx8 potentially protects cells from radiation treatment by eliminating ROS, which is linked to reduced levels of DNA damage and enhanced cell survival.

## 4. Discussion

Radiotherapy continues to be a central pillar of treatment for all solid tumour types, with over a third of PDAC patients receiving radiotherapy at some point during their disease course [23]. Unfortunately, radioresistance is one of the leading causes of poor prognosis in patients with PDAC, and as such, investigating the mechanisms underlying this radioresistance is crucial for the improvement of treatment strategies and patient survival.

ROS levels play an essential role in cell-cycle progression and proliferation [24]. Additionally, studies have shown how ROS are associated with apoptosis, metabolism, and hypoxic signalling [24,25]. ROS accumulation can give rise to oxidative stress, resulting in DNA damage and cell death [26]. Moreover, it is well known that ROS-mediated DNA damage is the primary source of cell death caused by radiotherapy [27]. Nevertheless, the cellular antioxidant defence system can help to regulate oxidative stress by reducing excess ROS and promoting DNA repair [28]. However, dysregulation within these defence systems can result in resistance to anti-cancer therapies [29,30].

Glutathione peroxidases (GPx) are a family of enzymatic antioxidants that play an essential role in ROS detoxification, particularly hydrogen peroxide (H_2_O_2_), using reduced glutathione (GSH) as its substrate [31]. Additionally, it is well-established that the GPx family protects cells from DNA damage caused by excessive ROS [32]. To date, eight different GPx family members (GPx1-GPx8) have been identified [33], and recent studies have demonstrated that several members of the GPx family play a crucial role in resistance to anti-cancer therapies by altering levels of oxidative stress [34,35,36]. GPx8 is a membrane protein located on the endoplasmic reticulum (ER) and is a molecular gatekeeper that plays a vital role in regulating H_2_O_2_, where the knockdown of GPx8 in HEK-293 cells encourages ER stress and decreased cellular viability [37]. Zhang et al. showed that GPx8 promotes migration and invasion, where high expression of GPx8 in lung cancer was correlated with a worse clinical outcome and prognosis [38]. Additionally, a recent study demonstrated GPx8 as a critical player in a metabolic-inflammatory pathway that acts as a robust regulator of cancer cell aggressiveness [39]. Despite recent research elucidating the different biological functions of GPx8, its role in regulating radiosensitivity in cancer remains largely unexplored.

Emerging evidence has demonstrated miRs as essential regulators of cancer initiation, promotion, progression, and resistance to anti-cancer therapies, including radiotherapy [40]. MiR-31 has been shown to act as either an oncogene or a tumour suppressor gene depending on the cancer type [41], and has been reported to be underexpressed in patients with PDAC [42]. Recent studies have revealed how miR-31 can influence invasion and migration in various cancers [43,44] and how it plays a vital role in regulating sensitivity to anti-cancer therapies [21,22]. However, its role in regulating radiosensitivity in PDAC remains to be investigated.

We showed that modulating miR-31 in PDAC cell lines can regulate radiosensitivity and the levels of DNA damage. Overexpressing miR-31 resulted in a reduction of DNA damage at 24 h post-radiation treatment; this may be explained by the promotion of DNA damage repair in the surviving cells or due to the failure of generating detectable gamma-H2A.X due to a large amount of cell death. Consequently, caspase 3/7 activity was measured as a marker of apoptosis to control for the discrepancy found between radiosensitivity and reduced DNA damage. We found that overexpressing miR-31 in BxPC-3 cells displayed substantial caspase 3/7 activity at 4 h and 24 h post-radiation treatment, indicating that the levels of gamma-H2A.X were difficult to detect and quantify due to the large amounts of cell death occurring at these time points. In comparison, suppressing miR-31 in Panc-1 cells displayed a significant reduction in caspase 3/7 activity 4 h and 24 h post-radiation treatment, indicating that suppressing miR-31 reduces the rates of apoptosis post-radiation treatment. Moreover, this may explain the differences observed within the accumulated cell counts recorded on day three and day six post-radiation treatment.

ROS have been demonstrated as critical regulators of radiosensitivity in cancer and are known to promote DNA damage and cell death. We analyzed H_2_O_2_ generation, a primary type of ROS in PDAC cells. We showed that H_2_O_2_ was elevated 20 min post-radiotherapy but was quickly returned to baseline by 4 h and 24 h post-radiotherapy when suppressing miR-31 in Panc-1 cells. By comparison, the H_2_O_2_ levels were significantly elevated at 20 min and 4 h post-radiotherapy when overexpressing miR-31 in BxPC-3 cells—indicating that cells with low miR-31 are better equipped at detoxifying ROS post-radiotherapy, thus promoting a radioresistant phenotype. Elevated levels of GSH are known to be associated with radioresistance by detoxifying excessive ROS [45], although we showed that levels of GSH remained unaltered across the PDAC cell lines, even post-radiotherapy; suggesting that miR-31 does not affect the GSH levels; therefore, playing no biological role in regulating miR-31-regulated radiosensitivity in PDAC.

**Figure 13:**
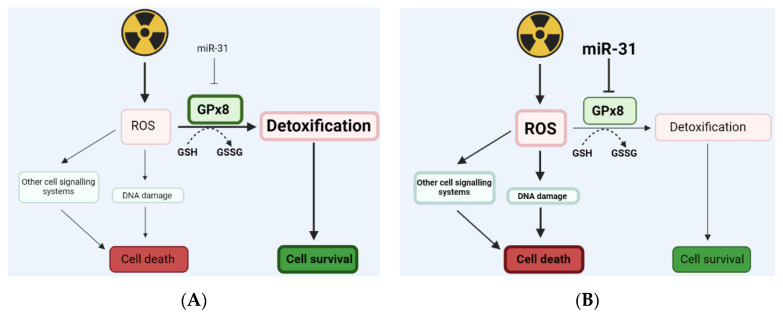
An illustration displaying how miR-31 may regulate levels of ROS by targeting GPx8. Radiation treatment can cause an increase in intracellular reactive oxygen species (ROS), which can target specific cell signalling systems and induce DNA damage directly, resulting in cell death. Glutathione peroxidase 8 (GPx8) is an enzymatic antioxidant that helps detoxify excessive ROS by converting reduced glutathione (GSH) into its oxidised form (GSSG), therefore promoting cell survival. (**A**) Low levels of miR-31 in PDAC cells may result in an increase in GPx8, supporting ROS detoxification and encouraging cell survival. (**B**) Whereas high levels of miR-31 in PDAC cells can potentially reduce GPx8. Moreover, reducing GPx8 can result in ROS accumulation, as ROS is being detoxified less efficiently, thus promoting cell death.

The mechanisms linking ROS and miR in regulating therapeutic resistance in PDAC are still unclear. However, using specific miRs for targeting antioxidant defence systems has been an area of thriving potential for improving cancer treatments. Pajic et al. presented miR-139-5p as a potent modulator of radiotherapy in breast cancer by targeting multiple DNA repair genes and ROS defence pathways [46]. MiR-17-3p has been revealed to target antioxidant enzymes, including GPx2, thus enhancing radiosensitivity in prostate cancer [47]. Furthermore, miR-153 was demonstrated to downregulate GPx1, leading to radioresistance in glioma stem cells [48]. Here, we show that miR-31 alters the expression of the antioxidant enzyme GPx8, where overexpressing miR-31 significantly reduces GPx8 levels, potentially resulting in a loss of its ability to detoxify ROS effectively, thus promoting DNA damage and cell death. However, suppressing miR-31 showed no significant increase in GPx8, despite detoxifying ROS effectively and displaying reduced DNA damage. The potential method by which miR-31 alters GPx8 and regulates radiosensitivity in PDAC cells is summarised in Figure 13.

Finally, we aimed to determine whether GPx8, independent of miR-31, contributed to PDAC radiosensitivity. We found that silencing GPx8 in the BxPC-3 parental cells enhanced radiosensitivity. Additionally, GPx8 expression protects cells from radiation treatment by detoxifying ROS more efficiently and is associated with reduced levels of DNA damage. These findings can be used for further research aimed at targeting antioxidants using miRNAs to improve the efficiency of radiotherapy for the treatment of PDAC.

This study has assessed miR-31’s influence on radiosensitivity in the in vitro PDAC cell models. Analyses of miR-31 expression in pre-treatment patient-derived tumour samples, stratified into good and poor response groups, would considerably add to the impact of this study. The patient-derived samples could be used to evaluate miR-31 and GPx8 expression as predictive biomarkers of responses to therapy.

## 5. Summary

To summarise, PDAC resistance to conventional therapies, including radiotherapy, remains challenging and is poorly understood. We demonstrated that miR-31 could represent a useful therapeutic target in PDAC by altering oxidative stress and DNA damage through the alteration of GPx8 expression, potentially restoring radiosensitivity. Screening patients for miR-31 expression status and thus personalized manipulation of the miRNA may promote an enhanced sensitivity to radiotherapy, improving patients’ overall survival.

## Data Availability

The microRNA target prediction algorithms can be found at TargetScan (http://www.targetscan.org/vert_72/), miRTargetLink (https://ccb-web.cs.uni-saarland.de/mirtargetlink/), and miRWalk (http://mirwalk.umm.uni-heidelberg.de/) accessed on 1 March 2021.
